# COVID-19–Associated Mold Infection in Critically Ill Patients, Chile

**DOI:** 10.3201/eid2705.204412

**Published:** 2021-05

**Authors:** Ricardo Rabagliati, Nicolás Rodríguez, Carolina Núñez, Alvaro Huete, Sebastian Bravo, Patricia Garcia

**Affiliations:** Pontificia Universidad Católica de Chile School of Medicine, Santiago, Chile

**Keywords:** SARS-CoV-2, COVID-19, coronavirus, 2019 novel coronavirus disease, severe acute respiratory syndrome coronavirus 2, zoonoses, coronavirus disease, viruses, COVID-19–associated invasive mold infection, Chile, respiratory infections, fungi

## Abstract

Patients with severe coronavirus disease (COVID-19) may have COVID-19–associated invasive mold infection (CAIMI) develop. We report 16 cases of CAIMI among 146 nonimmunocompromised patients with severe COVID-19 at an academic hospital in Santiago, Chile. These rates correspond to a CAIMI incidence of 11%; the mortality rate for these patients was 31.2%.

Invasive mold infection is a serious complication described in patients with severe viral pneumonia (*1*). Centers in Europe, China, and the United States have reported cases of fungal superinfections among patients with severe coronavirus disease (COVID-19). Aspergillosis is the main reported etiology; incidences range from 7.7% to 27.7% (*2*–*8*). Recently, the European Confederation on Medical Mycology and the International Society on Human and Animal Mycology published the diagnostic criteria for COVID-19–associated invasive pulmonary aspergillosis (CAPA), on the basis of histology, microbiology, imaging reports, and clinical factors (*9*).

We retrospectively identified adults admitted to the intensive care unit (ICU) at Hospital Clínico of UC-CHRISTUS Health Network in Santiago, Chile, during May 18–July 18, 2020 for COVID-19–associated invasive mold infection (CAIMI). We diagnosed CAIMI on the basis of respiratory failure, refractory fever, lung infiltrates, positive mold culture, positive galactomannan from serum, bronchoalveolar lavage (BAL), or a combination of these. The study was approved by the hospital’s institutional review board (ID no. 190320003, July 15, 2020).

We recorded clinical and microbiological data, imaging reports, treatments, and survival outcome. A thoracic radiologist (A.H.) reviewed chest radiographs and computed tomography (CT) scans, and we calculated a chest radiograph severity score (*10*). We confirmed fungal identification by matrix-assisted laser desorption/ionization time-of-flight mass spectrometry or sequencing.

Of the 856 COVID-19 patients admitted, 146 (17.1%) were hospitalized in the ICU and 16 (11%) received a diagnosis of CAIMI (Appendix Table). Median age of the 16 patients was 65 (range 30–89) years; 10 (62.5%) were male. Nine (56.3%) had hypertension, 4 (25%) asthma/COPD, 4 (25%) diabetes, and 3 (18.8%) obesity; none were immunocompromised. Median Acute Physiology and Chronic Health Evaluation (APACHE-II) score was 8 (range 4–20), and the median worst PaO_2/_FiO_2_ for each patient was 124 (range 57–476). Fourteen patients (87.5%) required invasive mechanical ventilation. In 12 cases (75%), prone position was applied for an average of 5 (range 2–19) days. All patients received antimicrobial drug therapy, 15 (93.8%) received corticosteroids, and 3 (18.8%) received tocilizumab.

We diagnosed CAIMI a mean of 18.5 (range 1–47) days after a positive COVID-19 test, at 14.5 (range 0–28) days after ICU admission, and at 12.5 (range 0–28) days after invasive mechanical ventilation was initiated. We performed BAL in 4 cases (25%); during bronchoscopy, we observed no ulcerative lesions in tracheobronchial mucosa. We diagnosed bacterial infection in 7 patients (43.8%). We obtained mold mycological evidence by fungal culture in 9 cases (56.3%) and galactomannan in 8 cases (50%); cultures came from tracheal aspirate in 7 cases and BAL in 2 (cases 15 and 16; Appendix Table). In 7 cases only 1 mold grew; in 2 cases >1 mold grew (cases 2 and 14). We identified a total of 12 molds: 9 (75%) *Aspergillus* spp. (4 *A. niger*, 2 *A. fumigatus*, 2 *A. terreus*, 1 *A. lentulus*), 2 (16.7%) *Rhizopus* spp. (1 *R. microsporus*, 1 *R. stolonifera*), and 1 (8.3%) *Scedosporium* spp. In relation to positive galactomannan, 6 cases (37.5%) were obtained from serum (index 1.29 [0.75–3.61]) and 2 (12.5%) from BAL (index 4.63 [3.65–5.6]).

All patients had chest radiographs, and 15 (93.7%) had CT. The mean radiologic score (*10*) at admission was 5 (range 3–6). Follow-up CT interpretation was challenging because of the presence of extensive viral pneumonia infiltrates. Findings included cavitation (case 11), nodules (case 16), cavitated nodule (case 15), pleural effusion (cases 3 and 14), pulmonary embolism (cases 4, 7, 11, 14, and 16), organizing pneumonia (cases 5 and 12), pneumothorax and bullas (cases 8 and 13) and preexisting airway disease (case 2). The mucormycosis patient (case 15) also had cerebral involvement shown by magnetic resonance imaging (Appendix Table).

Applying CAPA diagnostic criteria (*9*) to cases 1–13, we found 7 probable and 6 possible cases of CAPA. For the co-infection and non–*Aspergillus* identification cases, the CAPA criteria do not apply. Case 14 was in a previously healthy person who had *Aspergillus* and mucorales co-infection without other fungal foci. Case 15 was a proven disseminated mucorales infection, according to European Organization for Research and Treatment of Cancer/Invasive Fungal Infections Cooperative Group and the National Institute of Allergy and Infectious Diseases Mycoses Study Group criteria (*11*), in a man with uncontrolled diabetes (glycated hemoglobin 8.8%) who had *R. microsporus* identified in the airway and in an acute thoracic skin lesion, along with brain involvement suggested by cranial MRI. Case 16 involved 2 concurrent conditions and the fungal disease was limited to respiratory system. Considering the 16 CAIMI cases, we observed an incidence of 11% in patients with severe COVID-19 (6.8% aspergillosis, 0.7% mucormycosis, 0.7% aspergillosis/mucormycosis, and 0.7% scedosporiosis). Thirteen (81.3%) patients received antifungal therapy at standard doses: 10 (76.9%) received voriconazole, 2 (15.4%) liposomal amphotericin B, and 1 (7.7%) isavuconazole. We obtained therapeutic drug monitoring in 9 patients receiving voriconazole therapy (median 3.9 mg/L; range 0.1–7.2 mg/L). Eleven (68.8%) patients survived.

The 6.8% CAPA rate shown in our series is below the lower range reported previously (*2*–*7*). However, our retrospective design is a limitation for the real incidence calculations. Regarding the other molds identified, we have previously reported mucorales as the second most frequent identified mold in in our center (*12*), which is also the case in this report.

Diagnoses beyond aspergillosis, such as mucormycosis and scedosporiosis, add to a previously reported pulmonary fusariosis in an immunocompetent patient (*13*) and contribute to the knowledge of the epidemiology of fungal superinfections in severe COVID-19. Similarly, Garg et al. reported cases of mucormycosis in patients with severe COVID-19 (3 rhino-orbital, 3 pulmonary, 1 gastric, and 1 disseminated mucormycosis), with a mortality rate of 87.5% (*14*).

The classic predisposing underlying conditions associated with mold disease were absent in our cases. In fact, all patients were apparently immunocompetent, and the most relevant underlying conditions were the frequent conditions described in severe COVID-19 cases (*5*). Critically ill patients with COVID-19 have characteristics that could predispose them to fungal colonization and further invasive infection; these factors include severe hypoxia, broad-spectrum antibiotic drugs plus high corticosteroid doses, prolonged ICU length of stay, long intubation period, and airway/lung damage and infarction areas.

As previously reported (*4*), in our clinical series, 2 patients survived despite not receiving antifungal therapy. The explanation of this observation is not clearly understood. These patients could have had spontaneous mold clearance, favored at least in 1 case by less severe underlying conditions, lower steroid doses, not receiving tocilizumab, or minor lung injury. These cases might also have not been truly invasive infections but rather colonization, which illustrates the diagnostic difficulties in this field.

The certainty of CAIMI diagnosis is one of the main challenges in the COVID-19 scenario. Serum galactomannan is included in the CAPA diagnostic criteria (*9*), but this value has low sensitivity among patients without neutropenia (*15*). Another problem to consider is restrictions on performing BAL and bronchial or lung biopsy because of infection control policies and the conditions of severely ill patients. In our opinion, for CAIMI diagnostic criteria, it seems necessary to consider other factors, including host variables associated with the lung injury secondary to viral infection, bacterial superinfection, corticosteroids, thromboembolic disease, and others, that could favor a rapid and inadvertent progression from mold airway colonization to invasive infection.

In conclusion, we highlight the need for clinicians to have a high level of suspicion of mold infection in the list of possible superinfections among patients with severe COVID-19. In addition, CAIMI diagnostic criteria should include non-*Aspergillus* mold infections. 

Appendix Additional information on the characteristics of patients with COVID-19–associated mold infection, Chile.
