# Supplementary material for: COVID-19–Associated Mold Infection in Critically Ill Patients, Chile
Source: Emerg Infect Dis. 2021 May;27(5):1454–6. doi: 10.3201/eid2705.204412 (PMC8084475; doi:10.3201/eid2705.204412)
Supplement: Appendix — Additional information on the characteristics of patients with COVID-19–associated mold infection, Chile. [file 20-4412-Techapp-s1.pdf]

Article DOI: https://doi.org/10.3201/eid2705.204412

COVID-19–Associated Mold Infection in Critically Ill Patients, Chile

Appendix

Appendix Table. Characteristics of 16 COVID-19–associated mold infection (CAIMI) cases*

Case ID	Host and clinical factors: Age, y/sex	Concurrent conditions	Total corticosteroids (mg prednisone equivalent)	Tocilizumab	ICU days before CAIMI	IMV days before CAIMI	Imaging	Micrology evidence: Positive serum GM index >0.5	Positive BAL GM index ≥1	Positive culture from tracheal aspirate	Positive culture from BAL	CAPA diagnostic category†	Antifungal therapy	Voriconazole plasmatic levels (mg/L)	Outcome
1	56/F	Hypertension, obesity	780	Yes	28	28	Consolidation	NA	NA	*Aspergillus fumigatus*	NA	Possible	Voriconazole	5.2	Survived
2	71/F	COPD, hypertension	78	No	0	0	Consolidation	1.1	NA	*Aspergillus lentulus, A. niger, A. fumigatus*	NA	Possible	Voriconazole	7.2	Survived
3	30/F	Diabetes, hypertension	468	No	23	17	Consolidation, pleural effusion	NA	3.65	NA	NA	Probable	Liposomal amphotericin-B	NA	Died
4	57/M	COPD, hypertension	1,577.5	No	2	2	Consolidation, PE	1.7	NA	NA	NA	Probable	None	NA	Survived
5	76/M	None	576	No	18	15	Organizing pneumonia	NA	NA	*Aspergillus fumigatus*	NA	Possible	Voriconazole	3.4	Died
6	76/F	COPD, hypertension	104	Yes	26	22	Consolidation	3.61	NA	NA	NA	Probable	Anidulafungin‡	2.6	Died
7	48/M	Obesity	300	Yes	27	22	Consolidation, PE	NA	5.6	NA	NA	Probable	Voriconazole	2.8	Survived
8	68/M	None	None	No	15	11	Consolidation, pneumothorax	0.77	NA	NA	NA	Probable	None	NA	Died
9	73/F	Hypertension	500	No	15	13	Consolidation	NA	NA	*Aspergillus niger*	NA	Possible	Voriconazole	5.9	Survived
10	76/M	Hypertension	780	No	14	11	Consolidation	NA	NA	*Aspergillus terreus*	NA	Possible	Voriconazole	3.8	Survived
11	44/M	None	580	No	16	16	Consolidation, PE, cavitation	1.39	NA	NA	NA	Probable	Voriconazole	0.6	Survived
12	55/F	Obesity	570	No	10	8	Organizing pneumonia	0.75	NA	NA	NA	Probable	Voriconazole	Not done	Survived
13	72/M	COPD, diabetes	375	No	12	12	Consolidation, emphysema, bullas	NA	NA	*Aspergillus niger*	NA	Possible	Voriconazole	2.0	Survived
14	62/M	None	115.5	No	10	15	Consolidation, PE, pleural effusion	NA	NA	*Aspergillus niger, Rhizopus stolonifer*	NA	NA	None	NA	Survived
15	55/M	Atrial fibrillation, diabetes, hypertension	812	No	5	5	Consolidation, nodules, cavitated nodule	NA	NA	NA	*Rhizopus microsporus*	NA	Liposomal amphotericin-B	NA	Died
16	89/M	Asthma, diabetes, hypertension	None	No	7	0	Consolidation, PE, nodules	NA	NA	NA	*Scedosporium spp.*	NA	Isavuconazole	NA	Survived

*BAL, bronchoalveolar lavage; CAPA, COVID-19–associated invasive pulmonary aspergillosis; COPD, chronic obstructive pulmonary disease; GM, galactomannan; ICU, intensive care unit; IMV, invasive mechanical ventilation; NA, not applicable; PE, pulmonary embolism.
†CAPA diagnostic category column is based on Koehler P, Bassetti M, Chakrabarti A, Chen SCA, Colombo AL, Hoenigl M, et al. Defining and managing COVID-19-associated pulmonary aspergillosis: the 2020 ECMM/ISHAM consensus criteria for research and clinical guidance. Lancet Infect Dis. 2020 Dec 14 [Epub ahead of print]. https://doi.org/10.1016/S1473-3099(20)30847-1, and limited to cases in which only *Aspergillus* spp. were identified.
‡Antifungal therapy, case 6 was then changed to voriconazole.
